# Preventing Depression in Adults With Subthreshold Depression: Health-Economic Evaluation Alongside a Pragmatic Randomized Controlled Trial of a Web-Based Intervention

**DOI:** 10.2196/jmir.6587

**Published:** 2017-01-04

**Authors:** Claudia Buntrock, Matthias Berking, Filip Smit, Dirk Lehr, Stephanie Nobis, Heleen Riper, Pim Cuijpers, David Ebert

**Affiliations:** ^1^ Division of Online Health Training Innovation Incubator Leuphana University Lueneburg Lueneburg Germany; ^2^ EMGO+ Institute for Health and Care Research Department of Clinical, Neuro and Developmental Psychology VU University Amsterdam Amsterdam Netherlands; ^3^ Department of Clinical Psychology and Psychotherapy Friedrich-Alexander University Erlangen-Nuremberg Erlangen Germany; ^4^ Trimbos Institute Centre of Health-Economic Evaluation Utrecht Netherlands; ^5^ EMGO+ Institute for Health and Care Research Department of Epidemiology and Biostatistics VU University Medical Centre Amsterdam Netherlands; ^6^ Department for Gerontology University of Vechta Vechta Germany; ^7^ Institute of Telepsychiatry University of Southern Denmark Odense Denmark

**Keywords:** prevention, major depressive disorders, Internet, early intervention, cost effectiveness

## Abstract

**Background:**

Psychological interventions for the prevention of depression might be a cost-effective way to reduce the burden associated with depressive disorders.

**Objective:**

To evaluate the cost-effectiveness of a Web-based guided self-help intervention to prevent major depressive disorder (MDD) in people with subthreshold depression (sD).

**Methods:**

A pragmatic randomized controlled trial was conducted with follow-up at 12 months. Participants were recruited from the general population via a large statutory health insurance company and an open access website. Participants were randomized to a Web-based guided self-help intervention (ie, cognitive-behavioral therapy and problem-solving therapy assisted by supervised graduate students or health care professionals) in addition to usual care or to usual care supplemented with Web-based psycho-education (enhanced usual care). Depression-free years (DFYs) were assessed by blinded diagnostic raters using the telephone-administered Structured Clinical Interview for DSM-IV Axis Disorders at 6- and 12-month follow-up, covering the period to the previous assessment. Costs were self-assessed through a questionnaire. Costs measured from a societal and health care perspective were related to DFYs and quality-adjusted life years (QALYs).

**Results:**

In total, 406 participants were enrolled in the trial. The mean treatment duration was 5.84 (SD 4.37) weeks. On average, participants completed 4.93 of 6 sessions. Significantly more DFYs were gained in the intervention group (0.82 vs 0.70). Likewise, QALY health gains were in favor of the intervention, but only statistically significant when measured with the more sensitive SF-6D. The incremental per-participant costs were €136 (£116). Taking the health care perspective and assuming a willingness-to-pay of €20,000 (£17,000), the intervention’s likelihood of being cost-effective was 99% for gaining a DFY and 64% or 99% for gaining an EQ-5D or a SF-6D QALY.

**Conclusions:**

Our study supports guidelines recommending Web-based treatment for sD and adds that this not only restores health in people with sD, but additionally reduces the risk of developing a MDD. Offering the intervention has an acceptable likelihood of being more cost-effective than enhanced usual care and could therefore reach community members on a wider scale.

**Trial registration:**

German Clinical Trials Register: DRKS00004709; http://www.drks.de/DRKS00004709 (Archived by WebCite at http://www.webcitation.org/6kAZVUxy9)

## Introduction

Currently, major depressive disorder (MDD) is one of the leading causes of years lived with disability [1] and is further associated with substantial economic costs for society [2]. In high-income countries, the 12-month prevalence of MDD is estimated at 5.1% [3] with an annual incidence rate of 3% [4]. This implies that close to 60% of the depressed cases are new cases, which underscores the importance of both treatment and prevention of depression to reduce its disease burden. In fact, even when assuming full coverage of evidence-based treatments, approximately only one third of the disease burden could be averted [5,6]. In this context, attention has shifted to the prevention of MDD as an additional option to foster further reductions of depression’s disease burden by reducing the development of new cases.

Meta-analyses provide evidence for the effectiveness of psychological interventions to prevent first onsets and recurrences in depression [7-9]. Especially indicated prevention, targeting subthreshold symptoms of an emerging depression, appears to be particularly effective [8]. People are then screened for subthreshold depression (sD) not meeting the diagnostic criteria for a full-blown depressive disorder and are subsequently offered a preventive intervention. However, research on the cost-effectiveness of depression prevention is still limited. Economic evaluations conducted alongside randomized trials and economic evaluation studies using decision analytic modeling techniques suggest that preventive interventions for depression can be very cost-effective [10-13]. Research on how to deliver such interventions on a large scale to the community is scant. Using the Internet to provide community members with effective preventive interventions is currently viewed as a potentially cost-effective way of scaling up preventive interventions [14,15]. Web-based interventions are scalable. Scalability refers to the ability of the intervention shown to be effective in a research setting to be expanded under real world conditions. To reach a greater proportion of the eligible population while retaining effectiveness, only a small increase in therapeutic resources is required. Thus, the marginal cost per additional user get lower via an economies of scale effect. Economies of scale might also reduce variable costs (ie, therapist’s support per participant) because therapists become more efficient through better organization and experience.

To the best of our knowledge, no randomized controlled trial has investigated the cost-effectiveness of a Web-based guided self-help intervention to prevent the onset of diagnosed MDD. Elsewhere we reported the primary outcomes with respect to progression to MDD at 12 months [16]. Here we will evaluate the cost-effectiveness and cost-utility of the Web-based intervention among self-selected members from the community suffering from sD.

## Methods

### Study Design

We conducted and reported the health-economic evaluation in agreement with the Consolidated Health Economic Evaluation Reporting Standards statement [17] and the guidelines from the International Society for Pharmacoeconomics and Outcomes Research [18]. We conducted the economic evaluation with a 12-month time horizon from both a societal and a public health care perspective alongside a 2-armed pragmatic randomized controlled trial in Germany to establish the cost-effectiveness and cost-utility of an indicated Web-based guided self-help intervention in conjunction to usual care for people with sD as compared with enhanced usual care (ie, Web-based psycho-education in addition to treatment as usual). Whereas the societal perspective included all costs and consequences regardless by whom they were incurred, the health care system perspective only considers direct medical costs. Full details of the trial design can be found elsewhere [19]. The study was approved by the medical ethics committee of the University of Marburg (reference number AZ 2012-35K) and registered under DRKS00004709 in the German clinical trial registry.

### Participants

In total, 406 participants were included in the study. Participants were recruited from March 2013 to March 2014 from the general population via a large German health insurance company and through newspaper articles, on-air media, and related websites. We chose this open recruitment strategy as it reflects the clinical practice for this type of intervention, thus enhancing the trial’s ecological validity. As we conducted a pragmatic trial, the use of antidepressant medication was allowed as part of care-as-usual. However, participants needed to be on a stable dose for at least four weeks to be able to enter the study. Textboxes 1 and 2 present participant eligibility for inclusion and exclusion in the study.

**Inclusion criteria for the study.**

- Age 18 years and above
- Subthreshold depression (sD) (Centre for Epidemiological Studies Depression Scale (CES-D)≥16) as having some depressive symptoms not meeting the diagnostic criteria for a full-blown DSM-IV major depressive disorder (MDD) as assessed by the telephone-administered Structured Clinical Interview for DSM-IV (SCID)
- Internet access
- Informed consent

**Exclusion criteria for the study.**

- Meeting DSM-IV criteria for bipolar disorder or psychotic disorder
- Having a history of a major depressive disorder (MDD) in the past 6 months based on Kupfer’s model [20]

- Currently receiving psychotherapy for any kind of mental health problem
- Being on a waiting list for psychotherapy
- Having received psychotherapeutic treatment in the past 6 months
- Showing a significant suicidal risk (item 9 of the Beck Depression Inventory >1)

### Randomization and Masking

Randomization took place at an individual level and was conducted centrally by an independent statistician not otherwise involved in the study using an automated computer-generated random numbers table. Block randomisation, of size 2, was used to ensure equal sample sizes across both conditions. Details about the randomization procedure can be found elsewhere [16]. Study participants were not masked to their treatment allocation because of the nature of the intervention. SCID interviewers were, however, unaware of participants’ randomization status. Steps taken to maintain blinding are described in detail elsewhere [16]. In case of evidence for blinding breakdown, the interviewer was changed to the second outcome interview. The research staff conducting SCID interviews were not otherwise involved in the study.

### Procedures

All study participants had unrestricted access to care-as-usual (CAU). CAU for sD entails visits to the general practitioner (GP), but no treatment provided by mental health specialists. If depressive symptoms deteriorate, the German S3-Guideline/National Disease Management Guideline Unipolar Depression recommends psychotherapy and the prescription of antidepressant medication [21]. In our pragmatic study, we did not interfere in CAU. Instead, we maintained a naturalistic CAU condition to represent current clinical practice as far as possible. It should also be noted that health care use was measured in detail (see Measures), implying that we now can describe CAU in great detail (see Table 1).

**Table 1:** Mean annual per-participant costs (in €) by condition cumulative over the 12-month follow-up period (based on intention-to-treat sample, N= 406).

Cost categories		Intervention group, (n=202)	Control group, (n=204)	Incremental costs
		Mean (SD), €	Mean (SD), €	Difference, €
Intervention		299 (–)	10 (–)	289
**Health care costs**				
	General practitioner or internist	142 (142)	117 (154)	25	
	Mental health care	117 (308)	175 (447)	−58
	Other medical specialist^a^	243 (428)	236 (363)	7
	In-patient care	48 (679)	123 (1302)	−75
	Day care	0 (-)	35 (499)	−35
	Antidepressants	12 (41)	20 (57)	−8
**Patient and family costs**				
	Private therapist^b^	137 (354)	117 (246)	20
	Copayments^c^	27 (73)	30 (65)	−3
	Over-the-counter drugs	17 (34)	22 (42)	−5
	Informal care	323 (943)	384 (857)	−61
	Domestic help	143 (455)	120 (511)	−23
	Travel	29 (77)	28 (72)	1
**Productivity losses**				
	Absenteeism	1475 (2498)	1172 (2209)	303
	Presenteeism	1696 (1622)	2021 (2781)	−325
Total health care costs		904 (989)	768 (1777)	136
Total societal costs		4655 (4674)	4513 (5160)	143

^a^physiotherapist, occupational therapist.

^b^physiotherapist without prescription.

^c^patient’s contribution to prescribed medication.

#### Web-Based Guided Self-Help Intervention

The Web-based intervention, called GET.ON Mood Enhancer, is an online multimedia interactive intervention consisting of 6 sessions. Each session takes about 30 minutes to complete, but the amount of time spent on a session varies among users. Participants were advised to carry out at least one, preferably 2 lessons per week. Intervention usage was monitored by logfile analysis. On average, participants completed 4.93 of 6 sessions (Multimedia Appendix 1). The mean treatment duration was 5.84 (SD 4.37) weeks [16]. The intervention was developed by trained psychologists and therapists at the Leuphana University. Participants created their own password to access the intervention. Trial participants used the intervention free of charge. The intervention was based on behavioral activation and problem-solving therapy. The content of the intervention was frozen during the trial. An emphasis was placed on homework assignments to integrate newly acquired skills into daily life. Therefore, participants had the option to receive a set of about 42 standardized text-messages supporting them to integrate the learned techniques into their lives. Participants were also supported by an online-trainer, who provided written feedback after each session and monitored adherence to the intervention. In case of nonadherence, eCoach sent up to 3 reminders. The total time a trainer spent per participant was approximately 3 hours. Trained and supervised graduate students and health care professionals provided guidance. The guidance focused on supporting participants to work through the exercises. Trainers available on the Web did not provide therapeutic support. Further details about the intervention can be found in the study protocol [19].

#### Enhanced Usual Care

Participants in the control condition got access to an Web-based psychoeducational intervention, which was based on the German S3-Guideline/National Disease Management Guideline Unipolar Depression [21]. It informed participants about evidence-based treatments of depression should symptoms deteriorate. We thus mimicked and enhanced usual care because we provided patients with information that they may not always be offered thoroughly by their GP. Similar psycho-educational interventions have been shown to be effective in reducing depressive symptoms and is therefore suggested as a first-line intervention for early manifestations of depression in the primary care setting [22]. Participants could go through the materials as often as they want. However, we did not monitor its uptake and no additional support was provided to participants in the CAU condition.

### Outcome Measures

Self-reported measures (ie, EuroQol and SF-12) were collected at baseline, posttreatment (6 weeks after randomization), and 6- and 12-month follow-up using a secured Web-based assessment system (AES, 256-bit encrypted). The SCID interviews at baseline and 6- and 12-month follow-up were conducted by telephone.

#### Depression-Free Years (DFYs)

The main outcome in the cost-effectiveness analysis comprised DFYs. DFYs were based on the number of depression-free weeks up to the onset of a major depressive episode within the 12-month follow-up period. MDD was assessed according to DSM-IV criteria as assessed by the telephone-administered SCID [23,24] at 6- and 12-month follow-up covering the period to the previous assessment. Time to onset of MDD was assessed as accurately as possible using the Life Chart method as developed by Lyketsos [25]. In this method, life events were recalled using a calendar method where personal landmarks were used to determine the presence of depressive symptoms at each month during the follow-up period. During the interview, the first day of a depressive episode was established. If the exact day could not be established, the closest week (month) was defined and the mid-point of that week (month) was used. The interrater agreement in this study was substantial (Cohen kappa=0.77).

#### Quality-Adjusted Life Years (QALYs)

QALYs were used as the outcome in the cost-utility analysis. QALYs were based on the EQ-5D-3L (EuroQol) [26] and SF-6D (a subset of items of the SF-12v1) [27]. The EuroQoL and the SF-12 were assessed at baseline, posttreatment (6 weeks), and 6- and 12-month follow-up. The EQ-5D-3L comprises 5 items covering 5 domains (mobility, self-care, usual activities, pain/discomfort, and anxiety/depression), each of which is rated as causing “no problems,” “some problems,” or “extreme problems.” Theoretically, the EQ-5D-3L generates 243 different health states. Preference-based utilities for each of these health states are available for various countries with “full health” and “death” being anchored at 1 and 0, respectively. The SF-6D contains 6 dimensions (each with between 2 and 5 levels) and includes 6 items of the SF-12. The SF-6D generates 7500 different health states. Utility values were derived using Brazier’s algorithm [28,29]. QALY health gains were estimated by calculating the area under the curve (AUC) of linearly interpolated EQ-5D-3L and the SF-6D utilities to cover the whole follow-up period of 12 months. This method weighs the 12-month period by the respective utilities of each time period. A QALY gain of 1 would thus indicate full health throughout the 12-month trial period. For the main analysis, we used the EQ-5D-3L QALY based on the UK tariffs [30]. For the sensitivity analyses, we used the SF-6D QALYs because these are known to be more sensitive to changes in mild conditions [31].

### Resource Use and Costing

We used the Trimbos and iMTA questionnaire for costs associated with psychiatric illness (TiC-P) [32,33] for collecting data on health care utilization and productivity losses in patients with mental health conditions. We adapted the TiC-P for use in Germany and used it at baseline and at 6- and 12-month follow-ups. We computed health care costs, the patient’s out-of-pocket costs, the costs for informal care provided by the patient’s family and friends, and costs stemming from productivity losses due to absenteeism and lesser productivity while at work (presenteeism). Costs were expressed in Euro and indexed for the year 2013 (index factor 1.04) based on the German consumer price index [34]. Costs were converted to pound sterling (£) using the purchasing power parities reported by the Organization for Economic Cooperation and Development. For the reference year 2013, €1 was equated to £0.85.

#### Health Care Costs

We used 2 German guidelines for calculating health care costs [35,36]. A list of unit cost prices (ie, outpatient care) was used to compute the total health care costs on a per-participant basis [36]. Unit cost prices were as follows: (1) €20.92 for a visit to the GP, (2) €68.06 for an internal medicine consult, (3) €46.55 for a session with a psychiatrist, and (4) €81.44 for a session with a psychotherapist. Hospital stays were computed at €335.52 for an in-patient day in a mental hospital and €306.41 for an in-patient day in a hospital for psychosomatic medicine and psychotherapy. Costs were estimated by multiplying the units of resource use with corresponding unit cost prices.

#### Medication

The costs of prescribed medication were based on the German drug registry (Rote Liste) [37]. The basis for calculating costs of prescribed medication is the pharmacy retail price taking into account a specific pharmacy and manufacture’s discount. The rates of discount vary between private and statutory health insurances [36]. Therefore, we weighted the mean costs of the 3 largest packages with the same agent based on the daily defined dose by the statutory population share (89% of the German population are statutorily insured).

#### Intervention Costs

The total costs for the Web-based cognitive-behavioral intervention were estimated at €299 (£254) per participant by the provider (GET.ON Institute) including €180 for providing feedback by an eCoach. The total cost of the intervention was based on the actual market price of this intervention that has been determined by the provider. The GET.ON Institute aims to transfer scientific knowledge related to the present research into routine health care. This institute licenses the intervention under study from the Leuphana University, Lueneburg, to provide the intervention within routine preventive services of health insurance companies in Germany. We assumed that the cost of IT servers and infrastructure will increase if the intervention is scaled up because more servers are needed. However, these fixed costs are subject to an economies of scale effect (ie, marginal costs drop per additional user). The per-participant costs for the psycho-educational intervention were estimated at €10 (£9).

#### Patient and Family Costs

Out-of-pocket costs were directly obtained from participants. Costs for traveling were valued at €0.30 per kilometre. Opportunity cost (ie, time spent on the intervention) were valued at €23.10 per hour. Costs of informal care were valued using a shadow price of €18.33 per hour [36].

#### Costs of Productivity Losses

We followed the human capital approach to value productivity losses [38]. Productivity losses can be caused by days not worked (absenteeism) and by reduced efficiency while at work (presenteeism). Lost workdays due to absenteeism were valued at the corresponding gross average of participants’ income per day. Lost workdays due to presenteeism were computed by taking into account the number of work days for which the participant reported reduced functioning weighted by an inefficiency score for those days. Productivity losses from unpaid work (ie, domestic tasks) were valued using a shadow price of €18.33 per hour needed to pay for domestic help [36].

### Analysis

The study was powered to demonstrate a risk reduction of 10% between study conditions as statistically significant in a survival analyses with alpha<.05 (2-tailed), a power of (1−beta)=.80 using survival analysis, and accounting for a 20% dropout (calculated using PASS 12). However, the study was not powered to statistically test differences in health economic outcomes. Therefore, we took a probabilistic decision-making approach for our health economic inferences [39]. We did not discount costs and effects because the analysis was restricted to a 12-month follow-up period.

While evaluating the clinical outcomes, we reported all analyses in accordance to the CONSORT statement [40] (Multimedia Appendix 2). Data were analyzed on an intention-to-treat basis meaning that all participants were included in the analyses as randomized. To this end, we used Cox proportional hazard regression analyses to test differences in time to onset of MDD (in weeks) between intervention and control group. Concurrent use of antidepressants was included as covariate into the Cox proportional hazard model (post hoc). As the use of antidepressants was not a predictor of the outcome, it was excluded from the final model.

To account for missing data in cost and utility data, we used the regression imputation procedure in Stata version 13 (StataCorp) to obtain required predicted values. Predictors of outcome and dropout were identified by (logistic) regression analysis. Identifying predictors of outcome helped us to obtain the most likely values of the outcome whereas identifying predictors of dropout allowed us to correct for bias that might arise by differential loss-to-follow-up. We did not impute hospitalization costs because only 3 participants (0.7%) were hospitalized during the 12-month follow-up period leading to instable imputations. Therefore, we reassessed the impact of hospitalization costs on outcomes in a sensitivity analysis. At baseline, mean EQ-5D utility values were the same in intervention and control group (both groups: 0.74, SD=0.15). Therefore, no baseline adjustments were made when calculating QALYs. Differences in QALYs and DFYs between the intervention and control groups were assessed using independent samples *t*-tests.

In the cost-effectiveness analysis, the incremental cost-effectiveness ratio (ICER) was based on the incremental costs per unit of effect (DFY or QALY) gained. The corresponding equation is ICER=*(Costs*_INT_*– Costs*_CTR_*)/(Effects*_INT_*– Effects*_CTR_*)*, where *Costs* are the annual per-participant costs and *Effects* are the DFYs (QALYs) in intervention and control group (subscripted with INT and CTR, respectively). Sampling uncertainty in the ICER was handled using nonparametric bootstrapping by resampling patient-level data to generate 2500 simulations of the ICER. We bootstrapped the SURE model (seemingly unrelated regression equations; sureg command in Stata) to allow for correlated residuals of the cost and effect equations. Bootstrapping was used to obtain confidence intervals for cost-effectiveness ratios based on the percentile method, since parametric techniques are inappropriate for use on skewed variables and ratios. The bootstrapped ICERs were plotted on a cost-effectiveness plane with effects along the horizontal axis and costs along the vertical axis. A cost-effectiveness acceptability curve (CEAC) was graphed to assess the probability of the intervention being cost-effective at varying willingness-to-pay (WTP) ceilings. All analyses including uncertainty analyses were performed using Stata version 13.

#### Sensitivity Analyses

We tested the robustness of the outcomes of the main analysis in sensitivity analyses. First, the EQ-5D-3L might suffer from ceiling effects when measuring QALY changes in people with mild conditions. Therefore, we measured SF-6D QALYs, which have been reported to be more sensitive to QALY changes in milder conditions such as sD [31]. Second, we assessed the impact of in-patient care on the ICER because in-patient care is one of the main cost drivers and was surrounded by much uncertainty since only 3 hospital admissions occurred in the whole sample (0.7%, 3/406). Such outliers (driving costs in the control condition) could lead to misleading results, and these costs were removed in a sensitivity analysis.

## Results

### Sample Characteristics

In total, 406 participants were enrolled in the study (N_INT_=202; N_CTR_=204). At the posttreatment stage, 366 participants (90.1%, 366/406) were still participating. At 6- and 12-month follow-up, 325 (80.1%, 325/406) and 286 (70.4%, 286/406) participants completed the questionnaires, respectively. The CONSORT flowchart (of the participants through the trial) can be found elsewhere [16]. Dropout rates did not differ between experimental and control conditions except for the 12-month follow-up. Here, dropout was higher in the intervention group (χ^2^_1_ = 8.4, *P*=.004). Study dropout was not associated with baseline depressive symptom severity or any sociodemographic factor. Participants’ characteristics at baseline are presented in detail elsewhere [16]. In brief, the average participant was female aged 45 years with an above average level of education and employed.

### Effects

The mean depression-free survival time within the 12-month trial period was 43 weeks (95% CI 41-46) in the intervention group and 37 weeks (95% CI 36-40) in the control group [16], corresponding to 0.82 DFYs and 0.70 DFYs, respectively. The incremental effectiveness of 0.82−0.70=0.12 DFYs was statistically significant (95% CI 0.05-0.18; *t*_404_=3.37, *P*<.001). Between-group differences in EQ-5D, QALY gains were not statistically significant (intervention group: 0.78, SD=0.14 vs control group: 0.77, SD=0.13; *t*_404_=−0.99, *P*=.32), but incremental SF-6D QALY gains differed significantly between study groups (intervention group: 0.71, SD=0.08 vs control group: 0.67, SD=0.07; *t*_404_=−4.40, *P*<.001).

### Costs

At baseline, mean total costs were €483 (£411) in the intervention group and €528 (£449) in the control group, which is only a small difference of €45 (£38), indicating that randomization had been well balanced. Table 1 presents the 12-month accumulated per-participant costs for various cost categories by study condition. The mean health care costs were higher in the intervention group as compared with the control condition. This difference can largely be explained by differences in the costs of the intervention as compared with the control condition with Web-based psycho-education (€299 vs €10). Patient and family’s out-of-pocket costs and the costs stemming from changes in productivity losses remained quite similar between study groups. Mean total costs, as seen from both the health care and societal perspective, were hence slightly higher in the intervention group as compared with the control group (societal perspective: €143 (£121); health care perspective: €136 (£116).

### Societal Perspective

Table 2 shows the incremental cost, effects, and cost-effectiveness ratios (based on 2500 bootstrap simulations) for the main analysis and the sensitivity analyses and from both the societal and health systems perspective. From a societal perspective, the intervention resulted in a greater mean health benefit (0.12 DFY gained) achieved at higher mean total costs (€134; £114) as compared with enhanced CAU.

**Figure 1:**
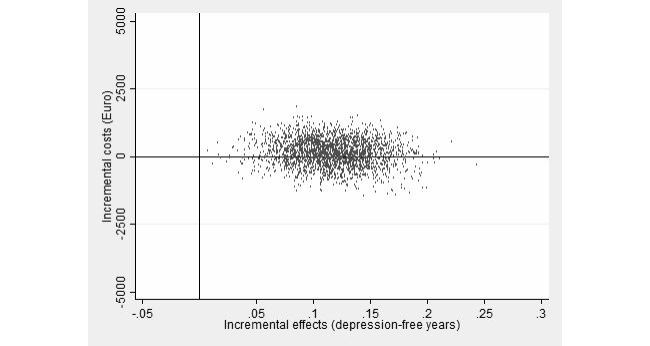
Scatterplot of 2500 replicates of the incremental cost-effectiveness ratio (mean differences in costs from a societal perspective and in depression-free years) on the cost-effectiveness plane: Web-based guided self-help intervention vs enhanced usual care.

The cost-effectiveness plane, representing the 2500 bootstrap replications, is shown in Figure 1. Most (62%) of the bootstrapped ICERs fell in the north-east quadrant, indicating a 62% probability that the intervention produces greater health, but at greater costs than enhanced CAU. The remaining 38% of ICERs fell in the south-east quadrant, indicating a 38% probability that the intervention dominates enhanced CAU because additional health gains are obtained for lesser costs. In other words, at a willingness-to-pay (WTP) of €0, the probability that the intervention must be regarded as more cost-effective than CAU is 38%.

**Figure 2:**
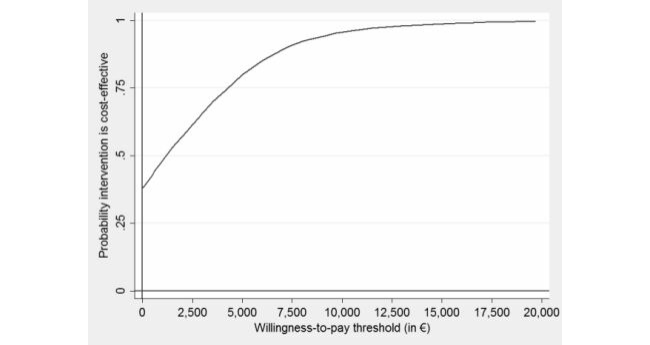
Cost-effectiveness acceptability curve showing the probability of the Web-based guided self-help intervention being cost-effective at varying willingness-to-pay ceilings (based on 2500 replicates of the incremental cost-effectiveness ratio using mean differences in costs from a societal perspective and depression-free years).

However, at a WTP of €7350 (£6248), €9680 (£8228), and €20,000 (£17,000) for gaining a depression-free life-year, the intervention’s probability of being more cost-effective than CAU rises to 90%, 95%, and 99% (Figure 2).

**Figure 3:**
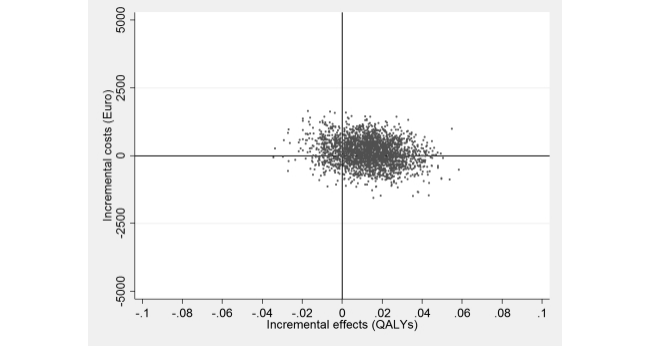
Scatterplot of 2500 replicates of the incremental cost-effectiveness ratio (mean differences in costs from a societal perspective and in quality-adjusted life years [QALYs]) on the cost-effectiveness plane: Web-based guided self-help intervention vs enhanced usual care.

The ICER based on QALY gains showed a small health benefit (0.01 QALYs gained) for higher mean costs (€134; £114). As seen in Figure 3, most of the simulated ICERs fell in the north-east quadrant (49%; see also Table 2).

**Figure 4:**
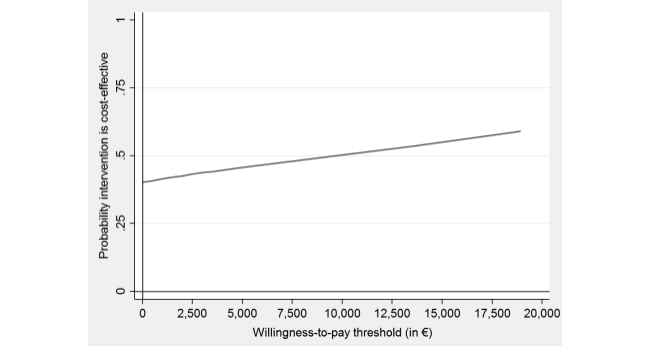
Cost-effectiveness acceptability curve showing the probability of the Web-based guided self-help intervention being cost-effective at varying willingness-to-pay ceilings (based on 2500 replicates of the incremental cost-effectiveness ratio using mean differences in costs from a societal perspective and quality-adjusted life years [QALYs]).

The intervention’s probability of dominating enhanced CAU was 35% when taking the societal perspective. Assuming a willingness-to-pay of €20,000 (£17,000) for gaining a QALY, this probability rose to 60% (Figure 4).

**Table 2:** Results of the main and sensitivity analyses (based on 2500 bootstrap simulations).

Analysis and perspective		Incremental	Incremental	Mean	Distribution over the ICER plane			
		costs, € (95% CI)	effects (95% CI)	ICER^a^ (95% CI)	North-east quadrant	North-west quadrant	South-east quadrant	South-west quadrant
**Cost-effectiveness, DFYs^b^**								
	Societal	134 (−827 to 1055)	0.12 (0.05 to 0.18)	1117 (−7546 to 11,737)	62%	-	38%	-
	Health care	135 (−146 to 418)	0.12 (0.05 to 0.18)	1125 (−1428 to 4715)	83%	-	17%	-
**Cost-utility, EQ-5D QALYs^c^**								
	Societal	134 (−827 to 1055)	0.01 (−0.01 to 0.04)	13,400^d^	49%	11%	35%	5%
	Health care	135 (−146 to 418)	0.01 (−0.01 to 0.04)	13,500^d^	68%	14%	16%	2%
**Sensitivity analyses**								
	SF-6D QALYs^e^, societal	134 (−827 to 1055)	0.03 (0.02 to 0.05)	4467 (−23,846 to 42,891)	60%	-	40%	-
	SF-6D QALYs, health care	135 (−146 to 418)	0.03 (0.02 to 0.05)	4500 (−5000 to 15,088)	83%	-	17%	-
**Without hospitalization costs**								
	Societal, DFY	233 (−649 to 1155)	0.12 (0.05 to 0.18)	1942 (−5169 to 14,705)	70%	-	30%	-
	Health care, DFY	245 (119 to 374)	0.12 (0.05 to 0.18)	2042 (865 to 5562)	100%	-	-	-
	Societal, EQ-5D QALY	233 (−649 to 1155)	0.01 (−0.01 to 0.04)	23,300^d^	13%	58%	3%	26%
	Health care, EQ-5D QALY	245 (119 to 374)	0.01 (−0.01 to 0.04)	24,500 (44220 to −22,333)	16%	84%	-	-

^a^ICER: Incremental cost-effectiveness ratio.

^b^DFYs: Depression-free years.

^c^EQ-5D QALYs: Quality-adjusted life years based on EuroQol.

^d^A dependably accurate 95% confidence interval for this distribution cannot be defined because there is no line through the origin that excludes alpha/2 of the distribution.

^e^SF-6D QALYs: Quality-adjusted life years based on SF-12.

### Public Health Care Perspective

From a public health care perspective, the health benefit (0.12 DFY gained) was also achieved at higher costs (€135; £115). In the cost-effectiveness plane, 83% of simulated ICERs fell in the north-east quadrant with the remaining 17% in the south-east quadrant (Table 2). The intervention’s probability to be more cost-effective than enhanced CAU increased to 51% at a WTP of €1250 (£1063) and became 90%, 95%, and 99% at a WTP of €3140 (£2669), €3,920 (£3332), and €6000 (£5100), respectively. From the societal perspective, the ICER from the public health care perspective based on QALY gains showed a small health benefit (0.01 QALYs gained) for higher mean costs (€135). In the cost-effectiveness plane, 68% of the simulated ICERs fell in the north-east quadrant (Table 2). The intervention’s probability of dominating enhanced CAU was 16% when considering health care costs. Assuming a willingness-to-pay of €20,000 (£17,000) for gaining a QALY, this probability was 64%.

### Sensitivity Analyses

Using the SF-6D resulted in a greater incremental QALY gain in favor of the intervention group (0.70 QALY, SD 0.08) as compared with the control group (0.67 QALY, SD 0.07), which was statistically significant (*t*_404_=4.40, *P*<.001). This agrees with available evidence that the EQ-5D suffers from a ceiling effect in less severe diseases [41]. At a WTP of €20,000 for gaining one QALY the probability of being cost-effective was 84% (societal perspective) and 99% (health care perspective). Hospital costs were higher in the control group so excluding these costs resulted in higher ICERs (Table 2). From a societal perspective and at a WTP of €20,000, the intervention’s probability of being cost-effective then became 53%.

## Discussion

### Principal Findings

Our study was set out to evaluate the cost-effectiveness and cost-utility of a Web-based guided self-help intervention to prevent the onset of MDD in adults suffering from sD in comparison with usual care enhanced with Web-based psycho-education. Main outcomes were DFYs and QALYs.

Significantly more DFYs were gained in the intervention group as compared with the control group. The probability that the intervention is deemed to be cost-effective depends on the willingness-to-pay (WTP) for a depression-free year. Assuming that the intervention is considered to be cost-effective if its likelihood of being cost-effective is greater than 90%, a WTP of €7350 and above for gaining a DFY would make the intervention cost-effective. While these DFY health gains were not mirrored in EQ-5D QALY gains, they were observed in SF-6D QALY gains with the latter being more sensitive to change in milder conditions such as sD and therefore not suffering as much from a ceiling effect as the EQ-5D does [31]. If society would be willing to pay €20,000 (£17000) for gaining a QALY, the probability of being cost-effective will be 64% for gaining an EQ-5D QALY and 84% for gaining a SF-6D QALY.

### Wider Context

Both burden of disease studies and cost of illness studies [1,2] attest to the importance of cost-effective interventions that can reduce the burden of depressive disorders on a wider scale. Recent meta-analytic evidence suggests that it is possible to prevent the onset of major depression using psychological interventions [8]. Results of our study showed that a guided eHealth intervention can successfully reduce the incidence of diagnosed major depression [16]. In addition, some evidence indicates that bibliotherapeutic self-help interventions for the prevention of depression represent good value-for-money [15]. However, economic evaluations in the field of depression prevention mainly relied on health-economic modelling [12,13,42] with direct evidence stemming only from 2 randomized controlled trials [10,11]. Although some trial-based economic evaluations of computerized cognitive-behavioral interventions for treating depressive symptoms exist (ie,) [43,44], to our knowledge, this is the first trial-based economic evaluation of a Web-based intervention to prevent the onset of major depression in an adult population with sD. Results from our trial adds to the converging evidence pointing to the effectiveness and cost-effectiveness (sometimes even costs-savings) of depression prevention across a range of outcomes.

### Limitations

This study has some limitations. First, the time horizon of this study was limited to 12 months. A recent meta-analysis showed a small positive association between effects of preventive interventions during the first months of follow-up, indicating that with passing months, the intervention effects get somewhat larger. However, meta-regression analyses suggested that the effects of interventions are lower at longer follow-up periods of 1 to 2 years [8]. Assuming diminishing long-term effects, the cost-effectiveness of this particular intervention will also decline. However, only few studies had longer follow-up periods than 2 years. Thus, more research with longer follow-up periods are needed to assess the long-term effectiveness and cost-effectiveness of preventive (Web-based) interventions. Second, we did not assess lifetime history of MDD at baseline, and therefore we cannot be sure if we prevented first-ever onsets of MDD or MDD recurrences. Future studies should thus clarify whether Web-based guided self-help interventions are cost-effective both for the prevention of first depression onset and the prevention of recurrence. Third, costs were assessed with the help of self-reports and may suffer from under-reporting. However, the structured questionnaire used in this study can be considered as valid instrument for recall periods up to 3 months [45]. Finally, the trial has been conducted in a highly-educated sample. Evidence suggests that better adherence is predicted by higher education [46]. In our trial, only 2% of participants were low educated. Hence, we cannot predict the uptake of such an intervention in less educated people or among people with a lower socioeconomic status. However, we used an open recruitment strategy in our trial mimicking the way how people will be recruited for eHealth interventions in the future, thus providing ecological validity to the sample on which this study is based. In other words, the trial sample reflects the population segments that are interested in engaging in a Web-based intervention. However, one conclusion drawn from this trial is that not all people who are in need of psychological interventions could be reached via the Internet. The applicability of Web-based interventions is related to (1) the acceptance of such interventions by the target population (ie, preferences for different treatment modalities, such as face-to-face interventions) and (2) the availability of technical requirements (ie, reliable access to the Internet).

### Clinical Implications and Future Research

Current guidelines on depression treatment (such as the NICE guideline and the Dutch Multidisciplinary Guideline for Depressive Disorder) recommend low-intensity psychosocial interventions (ie, computerized cognitive behavioral therapy) to manage (persistent) sD symptoms and mild-to-moderate depression [22]. Our study supports this recommendation by showing that an eHealth intervention may not only restore health in people with sD, but in addition reduces the risk of developing a major depressive disorder. Findings from our study also add that delivering cognitive-behavioral therapy over the Internet has a high probability of being cost-effective to prevent the onset of new depressive episodes. Given low participation rates in face-to-face preventive services and the potential to scale up Web-based interventions to efficiently alleviate the disease burden caused by MDD, it would be worthwhile to integrate such a Web-based intervention into routine practice. However, there are some opportunities and risks that need to be taken into account when scaling up this intervention. First, the feedback provided by an individual trainer on the Web in this Web-based intervention hinders scaling up the intervention. A recent review of randomized controlled trials showed that unguided interventions can also be effective (with lower adherence rates compared to guided interventions) [47]. To be more precise, in our study, out of the 202 participants who were initially assigned to the intervention, 138 (68.3%, 138/202) were intervention completers. This compares favorably with an unguided Internet-based intervention for the treatment of sD that was completed by 48.3% (49/102) of participants [48]. Providing guidance may not only affect the outcome and cost-effectiveness of the intervention but also the target group’s willingness to use such an intervention and thereby influencing the effects of such an intervention at population level. It is therefore not possible to predict the effects of this particular Web-based intervention at population level when it is offered without guidance by an eCoach. In addition, there are no guarantees that adherence and (by proxy) effectiveness will be maintained if Web-based preventive interventions are scaled up in the population. For example, Christensen et al reported that less than 1% of public registrants using a preventive intervention delivered openly on a website completed all modules [49]. Second, an unanswered question refers to how Web-based interventions could be rolled out to the population. For example, in the UK and the Netherlands, Internet-delivered cognitive behavioral therapy (CBT) is already prescribed by GPs [22]. In addition, Web-based interventions could be promoted independently at the GP’s (ie, promotion videos in the GP’s waiting rooms). However, a challenge in preventing depression is that most individuals at risk of developing a major depression do not show up in primary care. Therefore, innovative approaches are needed to reach these groups, for example through a systematic mental health screening of all people in specific settings (ie, occupational setting or universities) and to motivate those at risk to engage in preventive interventions (ie, acceptance facilitating interventions) [50]. However, such strategies do not guarantee uptake either. Third, the costs of the Web-based intervention were calculated without considering economies of scale effect. Economies of scale refer to the reduction in the cost per treatment of an intervention as a result of increasing the number of clients. Economies of scale arise because many of the costs associated with the Web-based intervention are fixed and not dependent on the number of clients (ie, hosting the intervention on a server) and thus increasing the intervention output reduces the fixed cost per treatment. Hence, we assume that the cost of IT infrastructure (ie, the per-client cost of servers to host the intervention) will be cheaper per additional client if the intervention is offered on a larger scale. Economies of scale might also reduce variable costs (ie, therapist’s support per participant) because therapists become more efficient through better organization and experience. However, the same technical resources available in the research setting (ie, reasonable Internet connections) may not be available when the intervention is scaled up. Finally, for some individuals a self-help approach might not be sufficient [51]. Some individuals may feel unable to apply psychotherapeutic self-help strategies. Some techniques could be inappropriately implemented by participants without guidance by an eCoach. One could argue that it is easier to observe and react to early signs of deterioration in face-to-face interventions as compared with Web-based interventions. Another potential negative effect of self-help interventions could be a delay in help-seeking leading to a further deterioration of symptoms, if the initial low-intensity self-help intervention should not be sufficient. Hence, multiple approaches to reach the target population are needed in successful depression prevention programs. Thus, future studies should evaluate the preventive effects of unguided Web-based interventions on the onset of MDD and compare the cost-effectiveness of unguided and guided interventions. In addition, implementation studies should be conducted to obtain real world effects of such interventions and to gather knowledge about the willingness to use these interventions in specific population segments (ie, in low-educated people, in a rural setting, or among people with lower socioeconomic status).

### Conclusions

Given the evidence for the efficacy of psychological interventions to prevent depression and the potential scalability and cost-effectiveness of Web-based interventions, large-scale dissemination of these interventions might be a promising strategy to alleviate depression’s disease burden in an affordable way and on a wide scale. However, before a nationwide dissemination could be considered, future studies need to evaluate the preventive effects of unguided Web-based interventions on the onset of MDD and compare the cost-effectiveness of unguided and guided interventions. Moreover, implementation studies are needed to obtain real world effects of such interventions and to gather knowledge about the willingness to use these interventions in specific population segments (ie, in low-educated people, in a rural setting, or among people with lower socioeconomic status).

## Appendix

Multimedia Appendix 1 Frequencies of the number of sessions completed.

Multimedia Appendix 2 CONSORT-EHEALTH checklist V1.6.2 [40].

## Abbreviations

CAU: care-as-usual
CES-D: Centre for Epidemiological Studies Depression Scale
DFY: depression-free year
DSM: Diagnostic and Statistical Manual of Mental Disorders
GP: general practitioner
ICER: incremental cost-effectiveness ratio
MDD: major depressive disorder
QALY: quality-adjusted life year
SCID: Structured Clinical Interview for DSM Disorders
sD: subthreshold depression
TiC-P: Trimbos and iMTA questionnaire for costs associated with psychiatric illness
WTP: willingness-to-pay

## References

[1] Vos T, Flaxman AD, Naghavi M, Lozano R, Michaud C, Ezzati M, Shibuya K, Salomon JA, Abdalla S, Aboyans V, Abraham J, Ackerman I, Aggarwal R, Ahn SY, Ali MK, Alvarado M, Anderson HR, Anderson LM, Andrews KG, Atkinson C, Baddour LM, Bahalim AN, Barker-Collo S, Barrero LH, Bartels DH, Basáñez MG, Baxter A, Bell ML, Benjamin EJ, Bennett D, Bernabé E, Bhalla K, Bhandari B, Bikbov B, Bin Abdulhak A, Birbeck G, Black JA, Blencowe H, Blore JD, Blyth F, Bolliger I, Bonaventure A, Boufous S, Bourne R, Boussinesq M, Braithwaite T, Brayne C, Bridgett L, Brooker S, Brooks P, Brugha TS, Bryan-Hancock C, Bucello C, Buchbinder R, Buckle G, Budke CM, Burch M, Burney P, Burstein R, Calabria B, Campbell B, Canter CE, Carabin H, Carapetis J, Carmona L, Cella C, Charlson F, Chen H, Cheng AT, Chou D, Chugh SS, Coffeng LE, Colan SD, Colquhoun S, Colson KE, Condon J, Connor MD, Cooper LT, Corriere M, Cortinovis M, de Vaccaro KC, Couser W, Cowie BC, Criqui MH, Cross M, Dabhadkar KC, Dahiya M, Dahodwala N, Damsere-Derry J, Danaei G, Davis A, De Leo D, Degenhardt L, Dellavalle R, Delossantos A, Denenberg J, Derrett S, Des Jarlais DC, Dharmaratne SD, Dherani M, Diaz-Torne C, Dolk H, Dorsey ER, Driscoll T, Duber H, Ebel B, Edmond K, Elbaz A, Ali SE, Erskine H, Erwin PJ, Espindola P, Ewoigbokhan SE, Farzadfar F, Feigin V, Felson DT, Ferrari A, Ferri CP, Fèvre EM, Finucane MM, Flaxman S, Flood L, Foreman K, Forouzanfar MH, Fowkes FG, Franklin R, Fransen M, Freeman MK, Gabbe BJ, Gabriel SE, Gakidou E, Ganatra HA, Garcia B, Gaspari F, Gillum RF, Gmel G, Gosselin R, Grainger R, Groeger J, Guillemin F, Gunnell D, Gupta R, Haagsma J, Hagan H, Halasa YA, Hall W, Haring D, Haro JM, Harrison JE, Havmoeller R, Hay RJ, Higashi H, Hill C, Hoen B, Hoffman H, Hotez PJ, Hoy D, Huang JJ, Ibeanusi SE, Jacobsen KH, James SL, Jarvis D, Jasrasaria R, Jayaraman S, Johns N, Jonas JB, Karthikeyan G, Kassebaum N, Kawakami N, Keren A, Khoo J, King CH, Knowlton LM, Kobusingye O, Koranteng A, Krishnamurthi R, Lalloo R, Laslett LL, Lathlean T, Leasher JL, Lee YY, Leigh J, Lim SS, Limb E, Lin JK, Lipnick M, Lipshultz SE, Liu W, Loane M, Ohno SL, Lyons R, Ma J, Mabweijano J, MacIntyre MF, Malekzadeh R, Mallinger L, Manivannan S, Marcenes W, March L, Margolis DJ, Marks GB, Marks R, Matsumori A, Matzopoulos R, Mayosi BM, McAnulty JH, McDermott MM, McGill N, McGrath J, Medina-Mora ME, Meltzer M, Mensah GA, Merriman TR, Meyer A, Miglioli V, Miller M, Miller TR, Mitchell PB, Mocumbi AO, Moffitt TE, Mokdad AA, Monasta L, Montico M, Moradi-Lakeh M, Moran A, Morawska L, Mori R, Murdoch ME, Mwaniki MK, Naidoo K, Nair MN, Naldi L, Narayan KM, Nelson PK, Nelson RG, Nevitt MC, Newton CR, Nolte S, Norman P, Norman R, O'Donnell M, O'Hanlon S, Olives C, Omer SB, Ortblad K, Osborne R, Ozgediz D, Page A, Pahari B, Pandian JD, Rivero AP, Patten SB, Pearce N, Padilla RP, Perez-Ruiz F, Perico N, Pesudovs K, Phillips D, Phillips MR, Pierce K, Pion S, Polanczyk GV, Polinder S, Pope CA, Popova S, Porrini E, Pourmalek F, Prince M, Pullan RL, Ramaiah KD, Ranganathan D, Razavi H, Regan M, Rehm JT, Rein DB, Remuzzi G, Richardson K, Rivara FP, Roberts T, Robinson C, De Leòn FR, Ronfani L, Room R, Rosenfeld LC, Rushton L, Sacco RL, Saha S, Sampson U, Sanchez-Riera L, Sanman E, Schwebel DC, Scott JG, Segui-Gomez M, Shahraz S, Shepard DS, Shin H, Shivakoti R, Singh D, Singh GM, Singh JA, Singleton J, Sleet DA, Sliwa K, Smith E, Smith JL, Stapelberg N, Steer A, Steiner T, Stolk WA, Stovner LJ, Sudfeld C, Syed S, Tamburlini G, Tavakkoli M, Taylor HR, Taylor JA, Taylor WJ, Thomas B, Thomson WM, Thurston GD, Tleyjeh IM, Tonelli M, Towbin JA, Truelsen T, Tsilimbaris MK, Ubeda C, Undurraga EA, van der Werf MJ, van Oppen J, Vavilala MS, Venketasubramanian N, Wang M, Wang W, Watt K, Weatherall DJ, Weinstock MA, Weintraub R, Weisskopf MG, Weissman MM, White RA, Whiteford H, Wiersma ST, Wilkinson JD, Williams HC, Williams SR, Witt E, Wolfe F, Woolf AD, Wulf S, Yeh P, Zaidi AK, Zheng Z, Zonies D, Lopez AD, Murray C, AlMazroa MA, Memish ZA (2012). Years lived with disability (YLDs) for 1160 sequelae of 289 diseases and injuries 1990-2010: a systematic analysis for the Global Burden of Disease Study 2010. Lancet.

[2] Luppa M, Heinrich S, Angermeyer MC, König H, Riedel-Heller SG (2007). Cost-of-illness studies of depression: a systematic review. J Affect Disord.

[3] Kessler RC, Sampson NA, Berglund P, Gruber MJ, Al-Hamzawi A, Andrade L, Bunting B, Demyttenaere K, Florescu S, de Girolamo G, Gureje O, He Y, Hu C, Huang Y, Karam E, Kovess-Masfety V, Lee S, Levinson D, Medina Mora ME, Moskalewicz J, Nakamura Y, Navarro-Mateu F, Browne MA, Piazza M, Posada-Villa J, Slade T, Ten HM, Torres Y, Vilagut G, Xavier M, Zarkov Z, Shahly V, Wilcox MA (2015). Anxious and non-anxious major depressive disorder in the World Health Organization World Mental Health Surveys. Epidemiol Psychiatr Sci.

[4] Ferrari AJ, Somerville AJ, Baxter AJ, Norman R, Patten SB, Vos T, Whiteford HA (2013). Global variation in the prevalence and incidence of major depressive disorder: a systematic review of the epidemiological literature. Psychol Med.

[5] Andrews G, Issakidis C, Sanderson K, Corry J, Lapsley H (2004). Utilising survey data to inform public policy: comparison of the cost-effectiveness of treatment of ten mental disorders. Br J Psychiatry.

[6] Chisholm D, Sanderson K, Ayuso-Mateos JL, Saxena S (2004). Reducing the global burden of depression: population-level analysis of intervention cost-effectiveness in 14 world regions. Br J Psychiatry.

[7] Barrera AZ, Torres LD, Muñoz RF (2007). Prevention of depression: the state of the science at the beginning of the 21st Century. Int Rev Psychiatry.

[8] van Zoonen K, Buntrock C, Ebert DD, Smit F, Reynolds CF, Beekman AT, Cuijpers P (2014). Preventing the onset of major depressive disorder: a meta-analytic review of psychological interventions. Int J Epidemiol.

[9] Biesheuvel-Leliefeld KE, Kok GD, Bockting CL, Cuijpers P, Hollon SD, van Marwijk HW, Smit F (2015). Effectiveness of psychological interventions in preventing recurrence of depressive disorder: meta-analysis and meta-regression. J Affect Disord.

[10] Smit F, Willemse G, Koopmanschap M, Onrust S, Cuijpers P, Beekman A (2006). Cost-effectiveness of preventing depression in primary care patients: randomised trial. Br J Psychiatry.

[11] Van't Veer-Tazelaar P, Smit F, van Hout H, van Oppen P, van der Horst H, Beekman A, van Marwijk H (2010). Cost-effectiveness of a stepped care intervention to prevent depression and anxiety in late life: randomised trial. Br J Psychiatry.

[12] Mihalopoulos C, Vos T, Pirkis J, Smit F, Carter R (2011). Do indicated preventive interventions for depression represent good value for money?. Aust N Z J Psychiatry.

[13] van den Berg M, Smit F, Vos T, van Baal PH (2011). Cost-effectiveness of opportunistic screening and minimal contact psychotherapy to prevent depression in primary care patients. PLoS One.

[14] Christensen H, Griffiths KM (2002). The prevention of depression using the Internet. Med J Aust.

[15] Mihalopoulos C, Vos T (2013). Cost-effectiveness of preventive interventions for depressive disorders: an overview. Expert Rev Pharmacoecon Outcomes Res.

[16] Buntrock C, Ebert DD, Lehr D, Smit F, Riper H, Berking M, Cuijpers P (2016). Effect of a web-based guided self-help intervention for prevention of major depression in adults with subthreshold depression: a randomized clinical trial. J Am Med Assoc.

[17] Husereau D, Drummond M, Petrou S, Carswell C, Moher D, Greenberg D, Augustovski F, Briggs AH, Mauskopf J, Loder E, ISPOR Health Economic Evaluation Publication Guidelines-CHEERS Good Reporting Practices Task Force (2013). Consolidated Health Economic Evaluation Reporting Standards (CHEERS)--explanation and elaboration: a report of the ISPOR Health Economic Evaluation Publication Guidelines Good Reporting Practices Task Force. Value Health.

[18] Ramsey SD, Willke RJ, Glick H, Reed SD, Augustovski F, Jonsson B, Briggs A, Sullivan SD (2015). Cost-effectiveness analysis alongside clinical trials II-An ISPOR Good Research Practices Task Force report. Value Health.

[19] Buntrock C, Ebert DD, Lehr D, Cuijpers P, Riper H, Smit F, Berking M (2014). Evaluating the efficacy and cost-effectiveness of web-based indicated prevention of major depression: design of a randomised controlled trial. BMC Psychiatry.

[20] Kupfer DJ (1991). Long-term treatment of depression. J Clin Psychiatry.

[21] Bundesärztekammer, Kassenärztliche Bundesvereinigung, Arbeitsgemeinschaft der Wissenschaftlichen Medizinische Fachgesellschaften (2011). German S3-Guideline/National Disease Management Guideline Unipolar Depression.

[22] (2010). Depression: The Treatment and Management of Depression in Adults (Updated Edition). NICE Clinical Guidelines, No. 90.

[23] Allen K, Cull A, Sharpe M (2003). Diagnosing major depression in medical outpatients: acceptability of telephone interviews. J Psychosom Res.

[24] Lyketsos CG, Nedstadt G, Cwi J, Heithoff K, Eaton WW (1994). The Life Chart Interview: a standardized method to describe the course of psychopathology. Int J Methods Psychiatr Res.

[25] Brooks R (1996). EuroQol: the current state of play. Health Policy.

[26] Gandek B, Ware JE, Aaronson NK, Apolone G, Bjorner JB, Brazier JE, Bullinger M, Kaasa S, Leplege A, Prieto L, Sullivan M (1998). Cross-validation of item selection and scoring for the SF-12 Health Survey in nine countries: results from the IQOLA Project. International Quality of Life Assessment. J Clin Epidemiol.

[27] Brazier JE, Roberts J (2004). The estimation of a preference-based measure of health from the SF-12. Med Care.

[28] Brazier J, Rowen D, Hanmer J (2008). Revised SF-6D scoring programmes: a summary of improvements. PRO Newslett.

[29] Dolan P (1997). Modeling valuations for EuroQol health states. Med Care.

[30] Brazier J, Roberts J, Tsuchiya A, Busschbach J (2004). A comparison of the EQ-5D and SF-6D across seven patient groups. Health Econ.

[31] van Straten A, Donker M, Tiemens B, Hakkaart-van Roijen L (2002). Manual Trimbos/iMTA questionnaire for Costs associated with Psychiatric illness (TiC-P).

[32] Bouwmans C, De Jong K, Timman R, Zijlstra-Vlasveld M, Van der Feltz-Cornelis C, Tan Swan S, Hakkaart-van Roijen L (2013). Feasibility, reliability and validity of a questionnaire on healthcare consumption and productivity loss in patients with a psychiatric disorder (TiC-P). BMC Health Serv Res.

[33] Destatis.

[34] OECD.

[35] Krauth C, Hessel F, Hansmeier T, Wasem J, Seitz R, Schweikert B (2005). [Empirical standard costs for health economic evaluation in Germany -- a proposal by the working group methods in health economic evaluation]. Gesundheitswesen.

[36] Bock JO, Brettschneider C, Seidl H, Bowles D, Holle R, Greiner W, König HH (2015). [Calculation of standardised unit costs from a societal perspective for health economic evaluation]. Gesundheitswesen.

[37] Rote Liste Service GmbH (2013). Rote Liste 2013.

[38] Drummond M, Sculpher M, Torrance G, O'Brian B, Stoddart G (2005). Methods for the Economic Evaluation of Health Care Programmes. 3rd ed.

[39] van Hout BA, Al MJ, Gordon GS, Rutten FF (1994). Costs, effects and C/E-ratios alongside a clinical trial. Health Econ.

[40] Eysenbach G (2011). CONSORT-EHEALTH: improving and standardizing evaluation reports of Web-based and mobile health interventions. J Med Internet Res.

[41] Lamers LM, Bouwmans CA, van Straten A, Donker MC, Hakkaart L (2006). Comparison of EQ-5D and SF-6D utilities in mental health patients. Health Econ.

[42] Lokkerbol J, Adema D, Cuijpers P, Reynolds CF, Schulz R, Weehuizen R, Smit F (2014). Improving the cost-effectiveness of a healthcare system for depressive disorders by implementing telemedicine: a health economic modeling study. Am J Geriatr Psychiatry.

[43] Warmerdam L, Smit F, van Straten A, Riper H, Cuijpers P (2010). Cost-utility and cost-effectiveness of internet-based treatment for adults with depressive symptoms: randomized trial. J Med Internet Res.

[44] Gerhards SA, de Graaf LE, Jacobs LE, Severens JL, Huibers MJ, Arntz A, Riper H, Widdershoven G, Metsemakers JF, Evers SM (2010). Economic evaluation of online computerised cognitive-behavioural therapy without support for depression in primary care: randomised trial. Br J Psychiatry.

[45] van den Brink M, van den Hout WB, Stiggelbout AM, Putter H, van de Velde CJ, Kievit J (2005). Self-reports of health-care utilization: diary or questionnaire?. Int J Technol Assess Health Care.

[46] Batterham PJ, Neil AL, Bennett K, Griffiths KM, Christensen H (2008). Predictors of adherence among community users of a cognitive behavior therapy website. Patient Prefer Adherence.

[47] Baumeister H, Reichler L, Munzinger M, Lin J (2014). The impact of guidance on Internet-based mental health interventions — A systematic review. Internet Interventions.

[48] Spek V, Nyklícek I, Smits N, Cuijpers P, Riper H, Keyzer J, Pop V (2007). Internet-based cognitive behavioural therapy for subthreshold depression in people over 50 years old: a randomized controlled clinical trial. Psychol Med.

[49] Christensen H, Griffiths KM, Korten AE, Brittliffe K, Groves C (2004). A comparison of changes in anxiety and depression symptoms of spontaneous users and trial participants of a cognitive behavior therapy website. J Med Internet Res.

[50] Ebert DD, Berking M, Cuijpers P, Lehr D, Pörtner M, Baumeister H (2015). Increasing the acceptance of internet-based mental health interventions in primary care patients with depressive symptoms. A randomized controlled trial. J Affect Disord.

[51] Ebert DD, Donkin L, Andersson G, Andrews G, Berger T, Carlbring P, Rozenthal A, Choi I, Laferton JA, Johansson R, Kleiboer A, Lange A, Lehr D, Reins JA, Funk B, Newby J, Perini S, Riper H, Ruwaard J, Sheeber L, Snoek FJ, Titov N, Ünlü Ince B, vanBastelaar K, Vernmark K, van Straten A, Warmerdam L, Salsman N, Cuijpers P (2016). Does Internet-based guided-self-help for depression cause harm? An individual participant data meta-analysis on deterioration rates and its moderators in randomized controlled trials. Psychol Med.

