# Supplementary figures and images for: Preventing Depression in Adults With Subthreshold Depression: Health-Economic Evaluation Alongside a Pragmatic Randomized Controlled Trial of a Web-Based Intervention
Source: J Med Internet Res. 2017 Jan 4;19(1):e5. doi: 10.2196/jmir.6587 (PMC5244034; doi:10.2196/jmir.6587)

Figure 2 Frequencies of the number of sessions completed

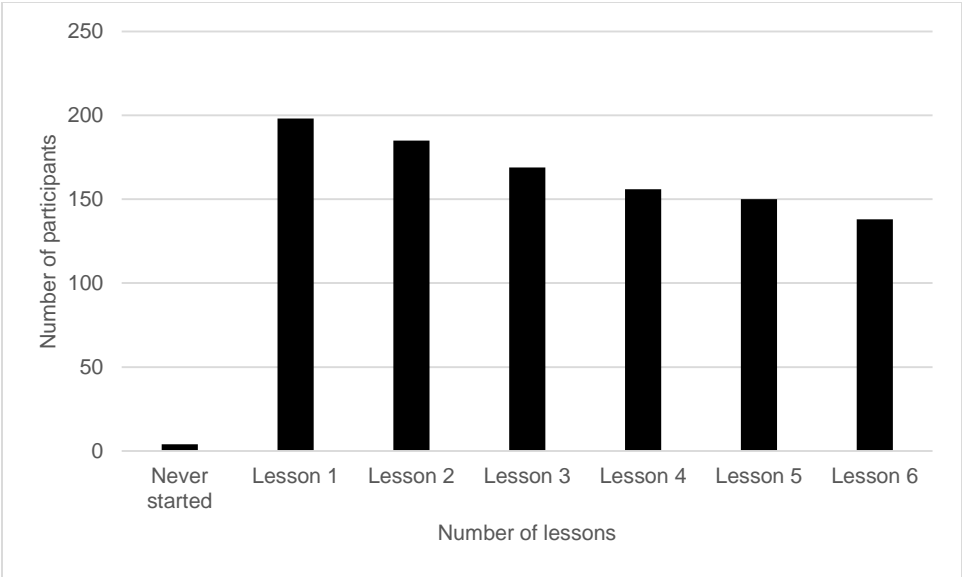

Supplement: Multimedia Appendix 1 [file jmir_v19i1e5_app1.pdf]
